# Domestication of Local Microbial Consortia for Efficient Recovery of Gold Through Top-Down Selection in Airlift Bioreactors

**DOI:** 10.3389/fmicb.2019.00060

**Published:** 2019-01-30

**Authors:** Ricardo Ulloa, Ana Moya-Beltrán, Camila Rojas-Villalobos, Harold Nuñez, Patricia Chiacchiarini, Edgardo Donati, Alejandra Giaveno, Raquel Quatrini

**Affiliations:** ^1^PROBIEN (CCT Comahue-CONICET, UNCo), Departamento de Química, Facultad de Ingeniería, Universidad Nacional del Comahue, Neuquén, Argentina; ^2^Microbial Ecophysiology Laboratory, Fundación Ciencia & Vida, Santiago, Chile; ^3^Facultad de Ciencias Biologicas, Universidad Andres Bello, Santiago, Chile; ^4^CINDEFI-CONICET, Universidad Nacional de La Plata, La Plata, Argentina; ^5^Millennium Nucleus in the Biology of the Intestinal Microbiota, Santiago, Chile

**Keywords:** *Acidithiobacillus*, acidophiles, domestication, adaptation, consortia, targeted metagenomics, metagenome derived assembly

## Abstract

Extreme acidophiles play central roles in the geochemical cycling of diverse elements in low pH environments. This has been harnessed in biotechnologies such as biomining, where microorganisms facilitate the recovery of economically important metals such as gold. By generating both extreme acidity and a chemical oxidant (ferric iron) many species of prokaryotes that thrive in low pH environments not only catalyze mineral dissolution but also trigger both community and individual level adaptive changes. These changes vary in extent and direction depending on the ore mineralogy, water availability and local climate. The use of indigenous versus introduced microbial consortia in biomining practices is still a matter of debate. Yet, indigenous microbial consortia colonizing sulfidic ores that have been domesticated, i.e., selected for their ability to survive under specific polyextreme conditions, are claimed to outperform un-adapted foreign consortia. Despite this, little is known on the domestication of acidic microbial communities and the changes elicited in their members. In this study, high resolution targeted metagenomic techniques were used to analyze the changes occurring in the community structure of local microbial consortia acclimated to growing under extreme acidic conditions and adapted to endure the conditions imposed by the target mineral during biooxidation of a gold concentrate in an airlift reactor over a period of 2 years. The results indicated that operative conditions evolving through biooxidation of the mineral concentrate exerted strong selective pressures that, early on, purge biodiversity in favor of a few *Acidithiobacillus* spp. over other iron oxidizing acidophiles. Metagenomic analysis of the domesticated consortium present at the end of the adaptation experiment enabled reconstruction of the RVS1-MAG, a novel representative of *Acidithiobacillus ferrooxidans* from the Andacollo gold mineral district. Comparative genomic analysis performed with this genome draft revealed a net enrichment of gene functions related to heavy metal transport and stress management that are likely to play a significant role in adaptation and survival to adverse conditions experienced by these acidophiles during growth in presence of gold concentrates.

## Introduction

Extreme acidophiles play an important role in the geochemical cycling of metals. They do so by affecting the solubility, speciation and precipitation of metals ions, either directly (e.g., by oxidation or complexation) or indirectly (through their influence on environmental redox conditions and pH). These capacities have long been harnessed in biomining practices (Harrison, 2016). One of such practice is the biooxidation of highly valued gold milled ores and their concentrates.

Gold is one of the ten scarcest elements in the Earth’s crust and is non-uniformly distributed (Reith et al., 2007). The metal occurs as solid inclusions within sulfide minerals or is finely dispersed in mineral crystal lattices (King, 2002). Pre-treatment procedures entailing the removal of mineral sulfides by the action of chemolithotrophic microorganisms are required to facilitate accessibility of extraction chemicals to the precious metal occluded in the mineral matrix. This process, known as biooxidation, has proven to be an economically viable, competitive and environmentally friendly biotechnology for the pre-treatment of refractory gold-bearing mineral concentrates (Rawlings et al., 2003).

Mineral concentrates are mostly processed in stirred tank reactors under relatively constant and homogeneous operative conditions (Rawlings, 2007). As a result, microbial communities recovered from operative tanks are low complexity assemblages of a sulfur- or iron-oxidizer and a mixo- or heterotrophic acidophile (Okibe et al., 2003; Norris, 2007; Bryan et al., 2011). Relevant acidophilic mesophiles involved in mineral biooxidation include the sulfur- and/or iron-oxidizing members of the *Acidithiobacillus* species complex (Nuñez et al., 2017), the iron-oxidizing leptospirilli, Gram-positive sulfobacilli and *Acidimicrobium* spp., and members of the archaeal genus *Ferroplasma* (Rawlings et al., 2003; Harrison, 2016). Relevant traits of effective mineral oxidizing communities recovered from tank reactors to date, are their ability to generate sulfuric acid or ferric iron which act as chemical lixiviants, and to endure the environmentally adverse conditions that develop during biooxidation.

Mineral dissolution achieved by the action of these microbes, changes the local environmental conditions (e.g., pH decrease; toxic metalloids concentration increase), triggering both community and individual level adaptive changes. Major physicochemical factors known to shape the phylogenetic structure of acidophilic microbial communities are the pH, the oxygen availability and the redox potential, all of which have been shown to influence spatial diversity patterns, as well as temporal successions (reviewed in Quatrini and Johnson, 2018). The influence of other stress factors [e.g., osmotic pressure (Suzuki et al., 1999)] and inhibitory compounds [e.g., the cyanide concentration (Harahuc et al., 2000)] on the adaptive evolution of acidic microbial communities are much less understood.

In addition to withstanding the evolving physicochemical conditions of bioleaching and the operative fluctuations inherent to the diverse industrial set-ups, any given effective mineral oxidizing community must be able to compete with native microbes present in the mineral ore or concentrate. In this context, considerations on the use of engineered (bottom-up approach) versus native consortia (top-down approach) have been raised (Rawlings and Johnson, 2007; Brune and Bayer, 2012). Even if designed consortia have proven useful in mineral dissolution in bioreactors at small scales (Okibe and Johnson, 2004), comparative leaching of cobaltiferous ore concentrates in stirred tank reactors have pointed to a superior performance by native microbial communities (Bryan et al., 2009, 2011).

Native microbial consortia colonizing sulfidic ores have been selected for their ability to survive under variable and adverse conditions (e.g., changing water availability), rather than for their ability to rapidly and efficiently oxidizing these ores under controlled conditions (Wakeman et al., 2008). Therefore, natural consortia cannot be expected to oxidize ores at maximum or even fairly efficient rates (Rawlings and Silver, 1995). Extended periods of acclimation and adaptation of these consortia to apparently similar target concentrates or ores seem to be required, before a reduction in the retention times, or an increase in growth and leaching rates is achieved (Rawlings and Silver, 1995; Rawlings, 2005; Rawlings and Johnson, 2007). Heavy metals (e.g., arsenic) seem to be relevant drivers in the adaptation native consortia to new mineral niches, acting as stringent selection forces against diversity (Barrick and Lenski, 2013). The microbes that are better adapted to the changing environmental conditions will outcompete the others. Alternatively, microorganisms may accumulate genetic changes that lead to improved fitness under the novel conditions.

The term ‘domestication’ has been used to describe the artificial selection of native species to obtain cultivated variants with desirable features and enhanced capacities to thrive in man-made environments (e.g., Gallone et al., 2018). Despite the fact that biomining is now established as a global biotechnology, little is known on the domestication of acidic microbial communities and their members. Complex patterns of domestication have been observed in various microbial species linked to human food production (Douglas and Klaenhammer, 2010), including gene gain, gene loss as well as high levels of horizontal gene transfer for specific survival traits (Makarova et al., 2006). All these events have also been reported for species of extreme acidophiles (Quatrini et al., 2016) and are likely associated to acclimatization and adaptation events taking place during long-term bioleaching processes.

In this study, high resolution targeted metagenomic techniques were used to analyze the changes occurring in the microbial community structure of local microbial consortia acclimated to growth under extremely acidic conditions and adapted to endure the conditions imposed by the specific target gold concentrate. In addition, after long-term domestication of the constituent microbes had been achieved, we evaluated key features of the dominant members of the consortium by means of metagenome sequencing and genome resolved analysis.

## Materials and Methods

### Sample Collection and Field Procedures

Eight samples were collected from the Andacollo gold mining district located in the semi-arid steep of northeastern Patagonia (37° 10′ 50.827′′ S, 70° 38′ 24.506′′ W; Neuquén, Argentina) in 2012. The average annual temperature and rainfall in the area is 13°C and 612 mm, respectively. Sampling sites (Supplementary Figure S1) were selected on the basis of the different history of management and use of the mine sites and the different characteristics of the input material (Supplementary Table S2). Selected sited included two mineral extraction galleries from the Buena Vista (BV) and San Pedro (SP) mines, from which slurry (S), water (W) and biofilm (B) samples were recovered, as well as two mine tailings, one active Relave Nuevo (RN) and another one abandoned Relave Viejo (RV), from which only slurry was sampled. Milled ore (O) with a particle size of approximately 1–5 mm was also sampled from the processing plant (PP). The refractory gold concentrate (RGC) was obtained from the flotation cells at the treatment plant of the Minera Andacollo Gold. Two kilograms of solid (milled mineral ore) and semisolid (slurry) samples taken from the upper layer (0–20 cm) of the target material and 1 L of water and 3 mL of biofilm samples were collected aseptically and stored at 4°C (for microbial consortia selection) and/or -20°C (for molecular analysis) for downstream processing. In all cases subsamples per site were pooled from the material collected from 4 equivalent independent points. Physicochemical parameters (pH, Eh, and conductivity) were measured *in situ* at the time of collection using a portable equipment (Orion Star 5, Thermo Scientific). Main elemental components (Fe, Mn, Zn, Pb, Cd, and Cu) were determined by atomic absorption following acid digestion (Merodio and Martínez, 1985).

### Acclimatization, Adaptation, and Domestication Experiments

Acclimatization to low pH, adaptation to the RGC and long term domestication of the best performing consortia were performed under controlled laboratory conditions at PROBIEN-UNCo (Neuquén, Argentina). For initial acclimatization to acidic operative conditions, batch cultures were setup in 125 mL Erlenmeyer flask containing 30 mL of fresh DSMZ 882 medium^1^ and 0K (9K without FeSO_4_) mineral salts medium (Silverman and Lundgren, 1959) and either ferrous iron (4 g/L FeSO_4_, pH 1.8) or sulfur (1% w/v, pH 3.0) as energy sources. In all cases the flasks were inoculated with 10% w/v or v/v of the Andacollo samples and incubated at 30°C with agitation (120 rpm) for 10 days. Microbial proliferation in each experiment was assessed directly by cell counts, and indirectly, on the basis of the iron and sulfur oxidation kinetics for the duration of the experiment. From these measures the growth latency phase, the production of protons, the percentage of the oxidized iron and the specific iron oxidation rate (in the exponential phase) were derived. Proton production was determined by volumetric titration with a NaOH 0.01 N solution (Merck) following standard procedures (Kolthoff and Sandell, 1963). Ferrous iron was quantified by spectrophotometry with the 1,10-phenantroline method (Vogel, 1989). Total iron was measured after reduction of all iron to the ferrous state with hydroxylamine hydrochloride as reducing agent; ferric iron was calculated as the difference between total iron and ferrous iron and percentages derived accordingly (Gomez et al., 1996). The specific iron and sulfur oxidation rates were calculated according to Franzmann et al. (2005).

Adaptation of the consortia to the RGC was carried out in 1 L shaken flasks containing 300 mL of DSMZ 882 or 0K media with additional energy sources (2 g/L FeSO_4_; 1% w/v S°; pH 1.8). A 3% w/v pulp density of microwave-sterilized RGC was used. The batch cultures were inoculated (at 5%) with a 1:1 mix of the iron- and sulfur-grown acclimated microbial consortia and incubated at 30°C with agitation (120 rpm) for 60 days and 4 subculturing rounds. As negative controls, equivalent amounts of the iron-grown and the sulfur-grown innocula were treated with 2% w/v thymol:methanol. Variations in pH, soluble iron and zinc concentrations were monitored every 3 days.

Domestication was pursued in a 10 L pneumatically agitated reactor with 0K medium operated in batch mode with reverse flow liquid recycling with a volumetric gas flow rate of 5 × 10^-5^ m^3^ s^-1^ and a 3% w/v pulp density as detailed in Giaveno et al. (2007). The reactor was inoculated (10% w/v) with the RGC acclimatized consortium in the absence of additional energy sources. Variations in the pH, the concentration of total iron, zinc and arsenic were determined periodically. The release of arsenic was measured with a commercial kit (Merck MQuant^TM^). Microbial proliferation was followed by direct cell counting in an optical microscope with phase contrast. Every 70 days (1 cycle) a sample of the evolving consortium was withdrawn from the reactor and used as inoculant for the next cycle. Between cycles, both the saline media and the RGC were replaced and the reactor was re-inoculated with the evolving consortia (at 10% v/v). The pH was maintained at 1.8 during the whole duration of the experiment and temperature was set at 30°C. Samples for molecular analysis (10 mL) were also collected at these points and the cell pellets stored at -80°C. Three samples (Cycles 0, 5, and 10) were selected for metagenomic analysis.

### DNA Isolation, Library Construction and Sequencing

Total DNA was extracted from the Relave Viejo Slurry (RVS, Supplementary Table S1) sample (10 g wet weight) using the E.Z.N.A^TM^ DNA Soil Extraction kit (Omega Bio-tek, Inc.) following the manufacturer’s instructions. Total DNA isolation from the cell pellets (0.5 g wet weight) were performed as in Nieto et al. (2009). DNA was resuspended in 100 μl TE buffer (Tris 10 mM, EDTA 1 mM, pH 8) and checked for integrity and quality using routine procedures. DNA concentrations were quantified with PicoGreen and a Synergy H1 microplate reader using a Take3^TM^ Micro-Volume Plate (BioTek Instruments, Inc.).

The amplification of the V4 region of the 16S rDNA was performed using the primers 515F and 806R (Caporaso et al., 2011). The reaction mixture (final volume 50 μL) consisted of 100 ng of DNA, 10 μM of each primer, 10 mM of dNTPs and 0.5 μL of Herculase II Fusion DNA Polymerase (Agilent). Amplification was performed under the following conditions: initial denaturation at 95°C for 2 min, 30 cycles at 95°C for 20 s, 55°C for 20 s, and 72°C for 30 s, and a final extension at 72°C for 3 min. After the amplification, purification of the PCR amplicons was accomplished using the QIAquick Gel Extraction Kit (Qiagen) and their concentration was determined with the PicoGreen^®^ kit (Turner BioSystems, Inc.). Amplicon library preparations were made using TruSeq DNA LT Sample Prep Kit (Illumina), following manufacturer’s instructions and library quality was evaluated using a Fragment Analyzer (Advanced Analytical). A total community DNA extract from the domesticated RVS consortium (Cycle 10) was nebulized to approximately 500 bp, and duplicate high quality libraries with insert sizes of ∼460 bp were prepared using Nextera^TM^ DNA Sample Preparation kit (Nextera, United States). Pooled libraries (at equimolar concentrations) were sequenced at the Genoma Mayor genomics facility (Santiago, Chile) using an Illumina MiSeq sequencer (250 bp paired-end reads).

Raw sequences generated in the present study were deposited at the National Center for Biotechnology Information (NCBI^2^) under the BioProject accession ID PRJNA499133. NCBI BioProject accession ID for the newly described draft metagenome assembled genome (RVS1_MAG) is PRJNA475418. Assembly and analysis of the remaining metagenomic data will be reported in future work.

### Sequence Manipulations and Bioinformatic Analyses

Amplicon sequence data were processed using Qiime2 v2018.8 (Bolyen et al., 2018) and filtering, denoising, forward and reverse merging, and chimera detection of the sequences was done with the computational workflow available through the open-source R package Divisive Amplicon Denoising Algorithm DADA2 (Callahan et al., 2016), using default parameters (maxN: 0, truncQ: 2, rm.phix: TRUE, maxEE: 2, chimera-method: consensus). Amplicon Sequence Variants (ASVs) were clustered at 100% identity. ASVs with only one representative (singletons) were removed from the data set for downstream bioinformaic analysis. Taxonomic assignment was done against the SILVA 16S rRNA database 132 (December, 2017 release) using vsearch (Rognes et al., 2016) incorporated in the q2-feature-classifier plugin algorithm (Bokulich et al., 2018). Statistical analysis was performed using Phyloseq (McMurdie and Holmes, 2013). The acidithiobacilli 16S rRNA gene sequences were further analyzed based on their placement on the guide tree reported previously by Nuñez et al. (2017) and their oligotype profiles derived (Eren et al., 2013).

Metagenomic sequence data from the domesticated consortium was preprocessed using Trimmomatic (v0.36) (Bolger et al., 2014). Reads with a >Q20 quality score were retained and assembled *de novo* using MetaVelvet (v1.2.10) (Namiki et al., 2012). The assemblies were binned using Megan5 by blastx against the nr database (August 2018 release). Bins matching publically available acidithiobacilli genomes (MOAD01, CP005986, NC_015850, LXQG01, LZYE01, LZYF01, LZYG01, LZYH01, NC_015942, NZ_LT841305, NZ_LT841305.1, CCCS02, MASQ01, LVZL01, NCBC01, LQRJ01, LVXZ01, QKQP01, PQJK01, NC_011761, NC_011206, MXAV01, LWSB01, LWSA01, LWSC01, LWRZ01, LWSD01, LWRY01, LZYI01, LGYM01, AZMO01, AFOH01, and AUIS01) were further analyzed using fragment recruitment with Bowtie2 (v2.2.6) (Langmead and Salzberg, 2012). Assemblies were merged using an in house script and manually curated to obtain consensus assemblies.

Draft assembly contigs >500 bp were used to calculate the average nucleotide identity index (ANIb) (Goris et al., 2007) with pyani (v0.1.3.2)^3^ and the *in silico* DNA-DNA hybridization index (DDH) was assessed using the Genome-to-Genome Distance Calculator with recommended formula 2 (Meier-Kolthoff et al., 2013) and species cutoff limits defined by Meier-Kolthoff et al. (2014).

For downstream bioinformatics analysis a preliminary annotation of the MAG recovered was performed with the RAST pipeline as described previously (Aziz et al., 2008). Genome comparisons were performed using the GET_HOMOLOGUES software package (v07112016) (Contreras-Moreira and Vinuesa, 2013). Orthology was determined based on all-versus-all Best Bidirectional BlastP Hit and COGtriangles (v2.1) as clustering algorithm. Pairwise alignment cutoffs were set at 75% coverage and *E*-value of 10E-05.

## Results and Discussion

### Study Rational and Experimental Design

**Figure 1:**
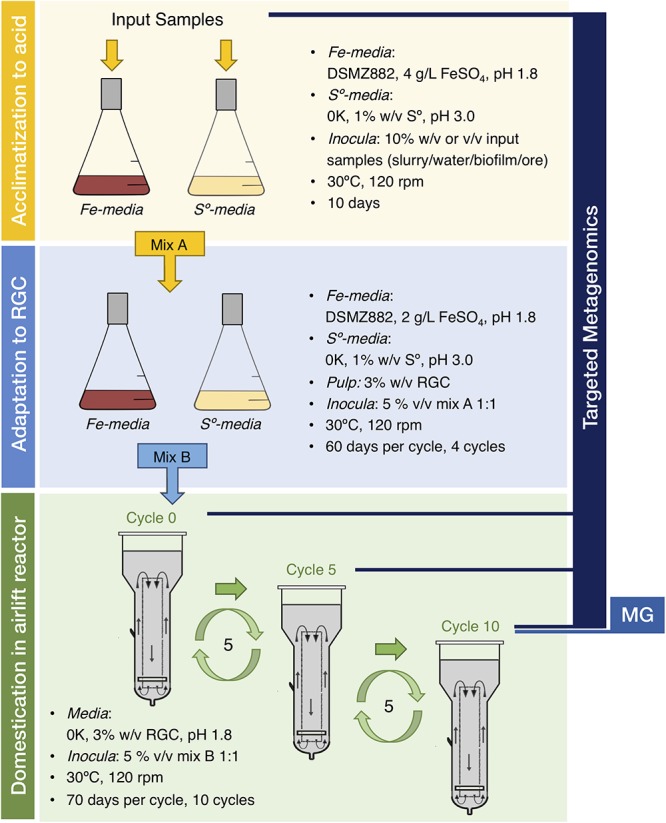
Top-down approach and experimental design utilized in the study.

To aid deciphering the underlying organizational principles and integrated physiological capacities of the microbial communities relevant in mining biotechnologies, we designed a long-term study focused on the biooxidation of gold-bearing mineral concentrates (Figure 1). We tested the “top-down approach” proposed by Rawlings and Johnson (2007), whereby indigenous microbial consortia (IMC) colonizing target sulfidic ores are selected for their improved mineral oxidation abilities under controlled conditions. Efficient biooxidation and pre-treatment of metal concentrates requires the microbial consortia to endure not only the extremely low pH of the leaching solutions, but also the intrinsically high loads of toxic metals and the organic flotation reagents used in the production of the concentrate (Rawlings, 2005; Hong et al., 2016). These factors, individually or collectively, may perturb microbial growth and trigger changes in both mineral associated IMC community composition and function, as reported in other types of mineral leaching setups (Edwards et al., 1999; Liu et al., 2014; Méndez-García et al., 2014). However, relevant drivers of community changes during top-down selection gold pretreatment have not been adequately defined. Thus, we exposed the Andacollo IMC to subsequent rounds of acclimation to acidic pH and adaptation to the target RGC, followed by an extensive domestication period under semi-industrial operational conditions to obtain microbial consortia with desirable features for RGC treatment. These features included improved relative iron oxidation capacities and greater relative acidogenic potential. Changes in the community structure of the IMC occurring along this process were assessed through targeted metagenomic and environmental metagenomic approaches, combined with genome resolved analysis.

The IMC suited for mineral pre-treatment were recovered from water, slurry, milled gold-bearing mineral ore and/or biofilm samples collected at the gold mine district in the semi-arid steep of northeastern Patagonia (Supplementary Figure S1 and Supplementary Table S1). Sampling points were selected on the basis of the different history of management and use of the mine sites and the different characteristics of the input material (Supplementary Table S2), as a proxy of the likelihood of finding active and adaptable microbial consortia. Some samples stood out for their high relative concentrations of Fe, Mn, and Mg (e.g., SPS, San Pedro Slurry), while others contained higher concentrations of Pb, Zn, and Cd (e.g., RVS, Relave Viejo Slurry). According to Inductively Coupled Plasma Atomic Emission Spectroscopy (ICP-AES) analysis, the RVS sample also contained arsenic (100 ppm) at concentrations comparable to those of the gold concentrate (114 ppm). These elements are generally enriched in target RGC obtained from the Andacollo mining area, primarily consisting of pyrite (FeS_2_), galena (PbS), sphalerite (ZnS) and arsenopyrite (FeAsS), and make this a potentially interesting consortia.

### IMC Acclimatization to Low pH, Adaptation to the RGC and Domestication

**Figure 2:**
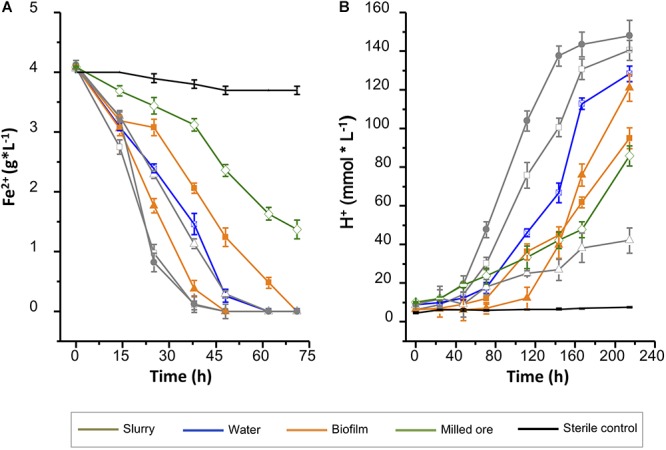
Acclimatization of IMC present in the Andacollo samples to acidic conditions at 30°C. **(A)** Oxidation of ferrous iron in DSMZ 882 medium. **(B)** Production of protons in 0K-S^0^ medium. Sample types are color coded to differentiate slurry (gray), water (blue), biofilm (orange), milled ore (green), and sterile control (black). *Symbols*: BVB (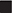); BVS (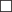); BVW (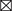); SPB (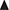); SPS (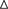); RVS (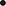); PPO (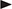); SC: sterile control (-).

To achieve the initial acclimatization of the IMC to acidic conditions batch cultures were setup as indicated in methods. Microbial proliferation in each experiment was assessed indirectly, on the basis of the net iron oxidation, the proton production and their respective specific activity rates (Figure 2). Iron oxidation capacity and acidogenic potential was higher in the slurry samples relative to the other sample types collected, regardless of the site of collection. Both capacities measured correlated with the conductivity of the samples, but appeared unrelated to the initial pH and Eh of the samples of origin (Supplementary Table S2). In the presence of iron as energy source the RVS, BVS (Buena Vista Slurry) and SPB (San Pedro Biofilm) samples had the shortest latency phases and the highest specific rates for iron oxidation (Figure 2A and Supplementary Table S3). Rust-colored precipitates were indeed observed at the time of sampling of the RVS and SPB samples, and considered biosignatures of iron oxidizers. When evaluated in 0K-S^0^ medium for acid production, the RVS and BVS consortia outperformed the SPB consortium, showing the highest acidification rates and the highest net proton production (Figure 2B and Supplementary Table S3). For these reasons, the RVS and BVS consortia were selected for scaling up and further adaptation to the RGC.

To adapt the IMC to the target concentrate batch experiments using 3% w/v pulp density and the best-performing acclimated IMC obtained above were set up and monitored every 15 days for the duration of the experiment (see section “Materials and Methods”). For each tested IMC the acclimatized consortia grown at acidic pH in the presence of iron or sulfur were mixed in a 1:1 proportion. Physicochemical changes (in pH, soluble iron and zinc concentrations), which occurred during the course of microbially-mediated mineral oxidation are shown in Table 1. After 60 days of incubation in the presence of the RGC only the RVS consortium showed persistent acidification of the media and efficient solubilization of metals (Fe and Zn) in both treatments in comparison with sterile controls. The BVS and BVW (Buena Vista Water) consortia lost viability after 3 passages and could not be rescued even in the absence of the concentrate (data not shown).

**Table 1:** Acidification and metal solubilization by three microbial consortia relative to sterile controls, after 60 days of adaptation to RGC.

	0K				DSMZ 882		
Consortia	ΔpH	Zn	Fe		ΔpH	Zn	Fe
RVS	0.93	8.78	47.1		0.33	3.6	1.96
BVS	0.34	2.87	3.79		0.38	2.59	1.56
BVW	0.52	2.52	3.50		0.34	2.32	1.35

*Total zinc (Zn) and iron (Fe) in solution (ppm) were measured using atomic absorption spectroscopy*.

**Figure 3:**
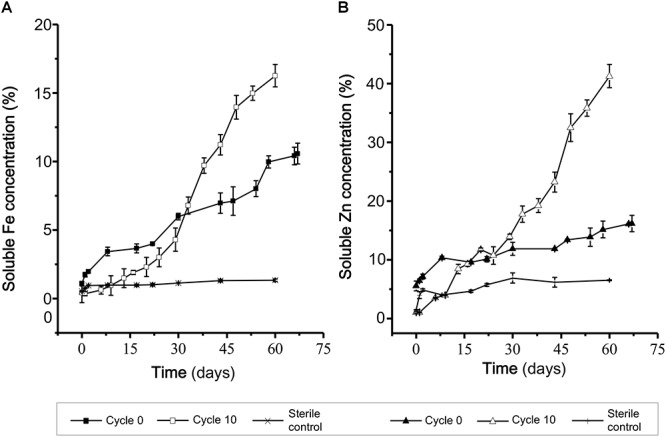
Biooxidation capacity of the adapted RVS consortium. **(A)** Biooxidation was assessed as the solubilization of Fe **(A)** and Zn **(B)** in DSMZ 882 medium at 30°C, during the Cycle 0 and Cycle 10.

For ICM domestication a 10 L airlift reactor was inoculated with 10% v/v of the pre-adapted RVS consortium and incubated at constant temperature and pH for 10 cycles of 70 days each. Samples were recovered upon initiation of the experiment (0 cycles), at a middle stage (5 cycles) and at a terminal stage (10 cycles) and stored for further analyses. Biooxidation capacity of the evolving consortia was monitored through the 70 days-cycles. Results obtained at the initial and final stages (Figure 3), showed clear differences in the biooxidation profile (Fe release days^-1^ 0.02 to 0.05 days^-1^ and Zn release 0.01 to 0.03 days^-1^) of the consortia evaluated, with an evident positive effect of long-term domestication of the consortia to the target mineral. The cycle 10 consortium showed a greater capacity to degrade the mineral concentrate with respect to the cycle 0 consortium, as reflected by the extent and the rate of solubilization of relevant cations (zinc and iron).

Improved performance achieved is indicative of an underlying long-term adaptive process, as observed also for designed consortia facing exposure to gold (Hong et al., 2016), copper (Hedrich et al., 2016), or chalcopyrite concentrates (Wang et al., 2014). However, the effect of long-term domestication has seldom been pursued [e.g., 40 weeks in (Wakeman et al., 2008) or 60 weeks in (Mutch et al., 2010)], regardless of the type of consortium considered (designed or native). Almost invariantly indigenous microorganisms are removed at the onset of the experiment (e.g., Mutch et al., 2010) or their subculturing history is not traceable (e.g., Spolaore et al., 2010), thus changes in the structure of the indigenous community and/or genetic changes in the community members are poorly understood.

### Global Changes in the Community Structure During Long-Term Domestication

To assess the changes elicited in the microbial community structure during domestication, the V4 region of the 16S rRNA gene was amplified and sequenced using the MySeq Illumina technology. Sequences obtained were clustered in Amplicon Sequence Variants (ASVs) at 100% identity using DADA2, which models and corrects Illumina-sequenced amplicon errors.

Rarefaction curves of the number ASVs as a function of the sampling effort are represented in Supplementary Figure S2. At sequencing depths greater than 1,000, all curves approached asymptotically to the maximum observed ASVs for each sample, except in the case of the un-adapted RVS consortium (RVSc), which did so only at greater sequencing depths. A decrease in diversity from the RVS, through adaptation cycles in the airlift reactor, to the domesticated consortium was apparent for all sequencing depths values. While the un-adapted RVS consortium harbored 471 ASVs (at 100% identity cutoff, >1× coverage), during adaptation to the RGC a steady decrease in total ASVs was observed, ending up with a domesticated consortium (Cycle 10) that harbored only 23.7% of the initial taxa (Table 2). The Shannon diversity index (H), Chao1 richness estimator (Ss) and Inverse Simpson dominance index (J) were calculated on the full dataset to assess this aspect further. The diversity and richness indices were significantly (*p* < 0.001) higher in the initial consortium than in the evolving consortia (Table 2), with a sharp drop in both indices at early stages of adaptation, consistent with a decrease in the compositional complexity of the assemblages along domestication. The domesticated consortium exhibited the overall lower number of ASVs, diversity, richness and evenness (Table 2).

**Table 2:** Diversity indexes for ASVs during domestication of the microbial consortia.

	Observed^a^	Chao1 (Se)^b^	Shannon (H)^c^	Inverted Simpson^d^
RVS	417	0	4.835	73.6
Cycle 0	162	0.249	3.864	51.5
Cycle 5	102	2.336	4	29.4
Cycle 10	99	4.642	1.7	2

*^*a*^Number of different taxa observed in the sample. ^*b*^Richness estimator. ^*c*^Biodiversity index. ^*d*^Dominance index.*

**Figure 4:**
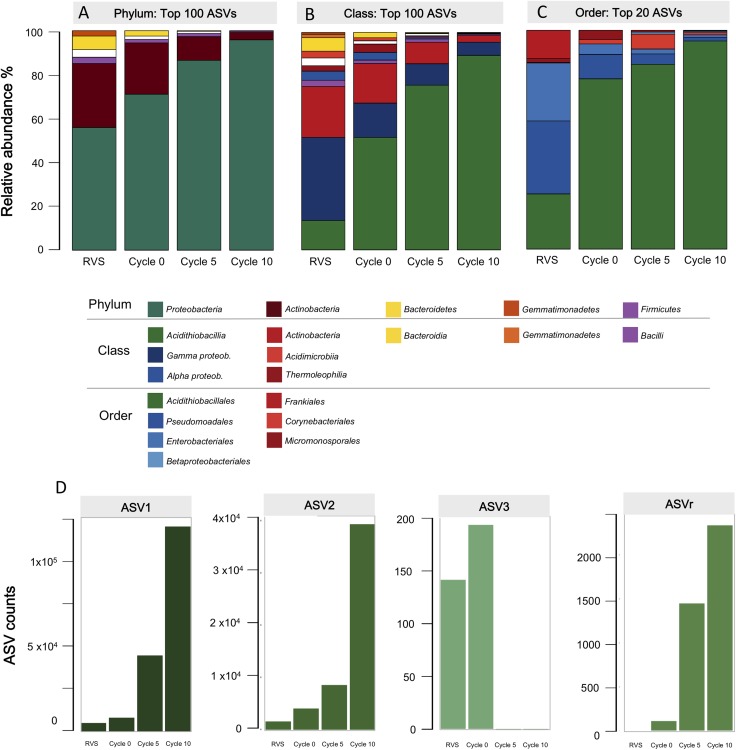
Taxonomic profiles and proportions of the RVS microbial consortium during adaptation cycles to the RGC. **(A)** Phylum level, top 100 ASVs; **(B)** Class level, top 100 ASVs; **(C)** Order level, top 20 ASvs. **(D)** Absolute abundance of the ASVs ascribed to the dominant taxon (*Acidithiobacillia*): ASV1 (184,713 total reads); ASV2 (51,844 total reads); ASV3 (191 total reads); ASVr (3,890 total remaining reads).

For downstream analyses the sequences obtained from each of the adaptation cycles were clustered at the 97% identity level to derive OTUs and the relative abundance of the microbial taxa across domestication cycles was assessed (Figure 4). An evident change in the dominance profile and complexity of the consortia was apparent from this data. At the phylum level (Figure 4A), the initial microbial community was mainly composed of typical soil Proteobacteria and Actinobacteria of the Terrabacteria group. From Cycle 1 onwards, the latter taxa were gradually outnumbered by the Proteobacteria, which reached a final abundance of >90% in the domesticated consortia. A comparison of the top 20 OTUs abundance at higher taxonomic levels (class/order) shows a highly uneven community structure across adaptation cycles (Figure 4B). During Cycle 1 the acidithiobacilli began to outnumber the other taxonomic orders (e.g., *Pseudomonadales*), with a neat dominance over the other microbes present at the end of the domestication process (from 15% in the RVSc to 90% at cycle 10) (Figure 4C). Seven out of nine total ASVs related to the obligate acidophilic genus *Acidithiobacillus* comprised this final population (Figure 4D and Supplementary Figure S3); three of these ASVs could be recognized from the beginning of the experiment. Sequence analysis indicated that ASV1 (76.7%), ASV2 (21.5%), and ASV3 (<1%), encompassing 99.7% of the total acidithiobacilli population, correspond to *A. ferrooxidans* species representatives (99% identity). During domestication the proportion of ASV1 and ASV2 populations increased, while low abundance ASV3 was negatively selected after 5 cycles. Dominance of *A. ferrooxidans*-like acidophiles over others, is likely related to the capacity of this species representatives to efficiently derive energy from the oxidation of the metal sulfides present in the gold concentrates (Rawlings et al., 1999), which in the case of the Andacollo RGC consists mostly of pyrite (FeS_2_), sphalerite (ZnS) and galena (PbS) (Supplementary Figure S4). It could also be related to the generalized capacity of members of this species to endure high concentrations of (heavy) metals (e.g., Fe, Zn, Au, Ag, Pb, and Cd) and metalloids (e.g., As).

### Temporal Variations in the Diversity of Acidophiles During Domestication

**Figure 5:**
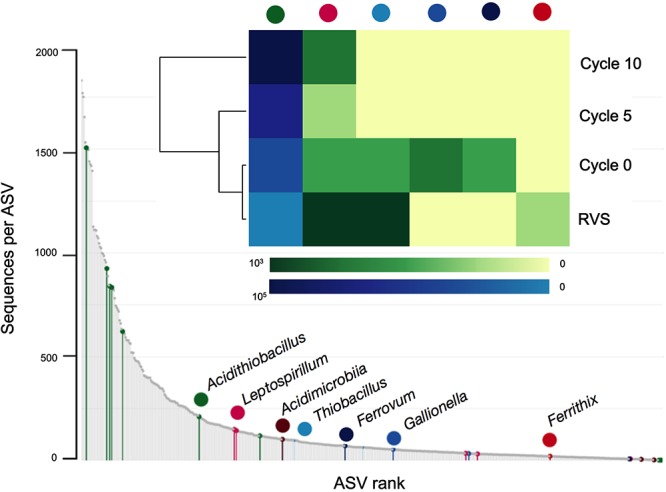
Rankabundance and heatmap of known acidophilic taxa during adaptation of the RVS microbial consortium. Values in heatmap are the actual read variant abundances of the top 2,000 ASVs.

The proportions of other acidophilic taxa were also assessed within and between adaptation cycles (Figure 5). Out of 13 genera of acidophiles included in the Silva 16S rRNA gene sequences database (v132) used for taxonomic assignment, seven were found to occur at some extent during domestication of the RVS consortium (5 of them at the initial sample and 2 in the final domesticated sample).

Two acidophilic taxa were present in all samples analyzed, implying they persisted across the domestication process; the dominant *Acidithiobacillus* and lower abundance *Leptospirillum* (6 ASVs in total) representatives, though their occurrence profiles varied both in total abundance and relative abundance per cycle (Figure 5). Four different general trends were observed across the data: (1) steady increase in abundance (*Acidithioacillus*), (2) steady decrease in abundance (*Ferrithrix, Thiobacillus*), (3) transient increase followed by disappearance at later adaptation cycles (*Ferrovum, Gallionella*) and (4) transient decrease followed by reappearance at later adaptation cycles (*Leptospirillum*).

All acidophiles detected are key players in the biogeochemical cycling of iron in low pH environments, being either ferrous iron oxidizers and/or ferric iron reducers (Hedrich et al., 2011; Johnson et al., 2012). Variations in the relative abundance of these taxa are likely related to fluctuations in the iron concentrations and the redox potential of the media occurring during domestication. Growth of *Ferrovum* spp. and *Gallionella* spp. have been seen to be inhibited in the presence of high iron concentrations (Tischler et al., 2013) and dominance of *Leptospirillum* spp. over *Acidithiobacillus* spp. occurs at high redox potentials (>690 mV) (Rawlings et al., 1999). Redox potential measured in the airlift reactor over the domestication cycles suggests conditions are more favorable to the acidithiobacilli. Also, both taxons have been described to endure high concentrations of metals and metalloids (e.g., As) and to proliferate efficiently in biooxidation tanks for gold concentrate pretreatment (Rawlings, 2005).

### Genetic Features of the Dominant ASVs in the RVS Consortium

To characterize the functional genetic potential of the domesticated RVS consortium (at Cycle 10) its metagenome was derived. The total sequence yield and quality indicators for the two replicate runs performed are shown in Supplementary Table S4.

**Figure 6:**
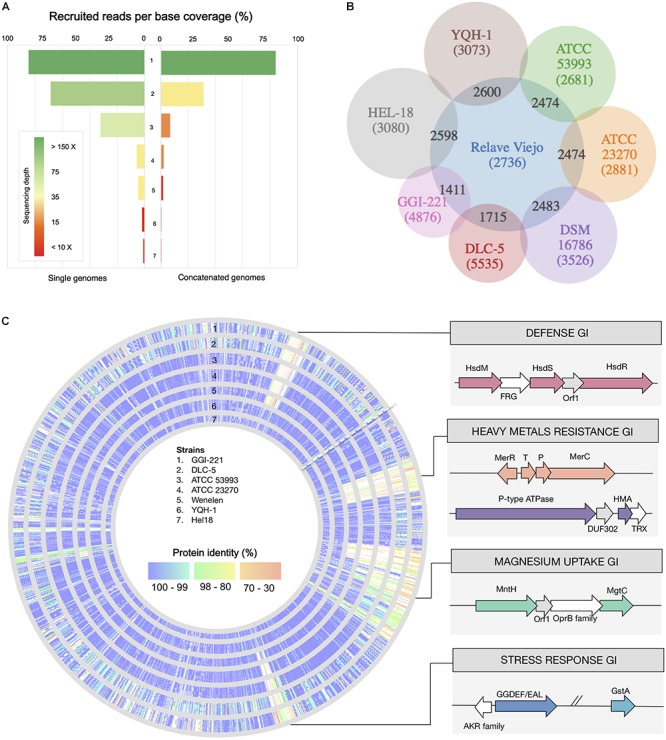
Domesticated RVS microbial consortium metagenomic analysis. **(A)** Per base coverage and sequencing depth coverage of different sequenced *Acidithiobacillus* spp. used as references (individually or concatenated). **(B)** Comparative analysis of shared vs. exclusive genes between the metagenome derived assembled genome (MAG) for the dominant taxon in the domesticated consortium (at Cycle 10) and sequenced *A. ferrooxidans* strains. **(C)** Genomic representation of the MAG_RVS1 showing conservation of gene products in sequenced *A. ferrooxidans* strains and gene islands (GIs) encoding adaptive functions.

Reads fulfilling quality filters (>Q20) were assembled *de novo* and their taxonomic affiliation assessed using Megan5. Among the contigs with assignable taxonomy, 92.5% could be ascribed to the acidithiobacilli and 3.9% to other microorganisms (3.6% remained unassigned). After fragment recruitment, per-base coverage of the *Acidithiobacillus* reference genomes varied between 2% (vs. *A. caldus*^T^) and 84% (vs. *A. ferrooxidans*^T^) and average redundancy coverage (depth) of the best represented acidithiobacilli (*A. ferrooxidans*^T^) was 675× (Figure 6A). A second *Acidithiobacillus* population (31.2% coverage, 33.8 × depth) matching *A. ferridurans* JCM 18981, was also identified. These results are consistent with the ASVs populations identified through 16S rRNA V4 targeted metagenomic analysis during domestication of the RVS consortium (see above).

The largest sequence bin recovered was assembled against the cognate reference genome and compared to individual and combined *de novo* assemblies obtained using all the reads generated. The metagenome derived assembly obtained using this data (RVS1_MAG) has been deposited in GenBank database under the BioProject accession number PRJNA499028. Total read recruitment against this MAG indicates that this strain (or a population of strains) encompasses the 74.97% of the total sequence trimmed, qualifying as the dominant domesticated population.

The RVS1_MAG assembly produced is 2.83 Mb long and is based on 1.4 Gbp of Illumina data, which provides an average 359 fold coverage of the genome. The draft genome consists of 24 contigs ≥ 10,000 and 25 smaller contigs. This MAG is predicted to be 99.9% complete (Raes et al., 2007) and encodes 3,078 protein coding genes and 46 RNA genes. Its average G + C content of the RVS1 genome is 58.8% and its average nucleotide identity against the *A. ferrooxidans* ATCC 23270^T^ (NC_011761) is 99.69% (ANIb), indicating that RVS1 is indeed a representative of the *A. ferrooxidans* species. Gene content comparative analysis against sequenced representatives of the species (Figure 6B) indicated that approximately 70% of the RVS1_MAG predicted genes have a conserved ortholog in all *A. ferrooxidans* strains analyzed. Average protein identity (at the amino acidic level) for this set of core genes is 99.7%, as expected for closely related microorganisms. Genes related to energy metabolism that are diagnostic of the species [e.g., *rus, pet*, *cox*, etc. (Quatrini et al., 2009)] fell in this category. A set of 337 to 658 partially shared gene (present in 1 to 6 out of the strains under comparison), having as well higher average levels of amino acidic sequence divergence (94.6–99.5% identity) and an additional 400 highly diverged gene products (62.2–97.5% identity), made up this MAGs flexible genome (Supplementary Table S5). Mapping of the gene orthologs present in the analyzed *A. ferrooxidans* strains is shown in Figure 6C.

Most of RVS1’s exclusive genes were hypotheticals or had no predicted function. Partially shared genes (present in only one or two strains, besides the MAG) and most differentiated genes (genes sharing less than 80% sequence identity) included transporters for several metals (e.g., manganese and iron), heavy metals (e.g., cadmium cobalt, lead, and zinc) and removal of other toxic compounds (e.g., mercury). Given the characteristics of the RGC, enriched in lead and zinc containing mineral sulfides and sulfates (galena and anglesite), the presence in the assembled MAG of 8 copies of gene encoding the P-type ATPases involved in cellular detoxification, and conferring resistance to lead, zinc and other heavy metal cations, is of note. Also, restriction modification systems, and stress response and management related genes concurred with genes that are typically horizontally transferred (e.g., conjugation genes, transposases, and recombinases) in what appeared to be strain specific mobile genetic elements or MGEs (Figure 6C). These functions could have a significant role in adaptation and survival to adverse conditions experienced by these acidophiles during growth in presence of gold concentrates.

## Conclusion

The top down approach pursued in this work proved to be a successful strategy to select and adapt an effective mineral oxidizing community for the pretreatment of RGCs originating from the Andacollo gold mineral district, and potentially also for gold concentrates of similar mineralogy and characteristics. Key to this achievement, were the comprehensive sampling of several different mine sites and types of input material, the sequential acclimatization to low pH, and the long-term adaptation to the target mineral concentrate. Through this processes the RVS local microbial consortium, obtained from an old mine tailing on site, was domesticated to the RGC, i.e., selected for its ability to survive under the specific polyextreme conditions imposed by the acidic, oxidant and highly toxic settings evolving during biooxidation of the gold mineral concentrate. Reasons behind the adequacy of the RVS slurry sample to provide a microbial consortia that could withstand the evolving physicochemical conditions imposed by the experimental setup used, seem to be the comparable and relative high concentrations of heavy metals (Pb, Zn, and Cd) in the input material. Acidophiles colonizing microniches in this slurry may have thus been pre-adapted to the characteristics of the gold concentrate targeted. Based on this evidence, one practice that the biomining industry should take in consideration to achieve sustained or improved metal recoveries is to select and maintain indigenous microbial consortia with desirable features emerging from their pre-adaptation to specific target minerals and enhanced capacities to thrive in man-made environments originating from domestication under controlled conditions.

The domestication process evaluated in this study entailed changes, from the input sample, through the adaptation cycles, to the final RVS consortium, in both structure and biooxidation capacity of the community. Results obtained using sensitive directed metagenomic techniques indicated that the operative conditions evolving through biooxidation exerted strong selective pressures that, early on, purged the biodiversity in favor of a few dominant members of the genus *Acidithiobacillus* over other iron oxidizing acidophiles. Metagenomic analysis of the domesticated consortium achieved at the end of the adaptation experiment enabled reconstruction of the RVS1-MAG, a novel representative of *A. ferrooxidans* from the Andacollo gold mineral district. Comparative analysis performed with this draft genome and available *A. ferrooxidans* genomes from diverse geographical origins and industrial settings, revealed a net enrichment of gene functions related to heavy metal transport and stress management that are likely to play a significant role in adaptation and survival to adverse conditions experienced by these acidophiles during domestication. Specific characteristics conferring to the RVS consortium with improved biooxidation capacities await future experimental evaluation and are conditioned to isolation in pure culture of the dominant *Acidithiobacillus* ASVs (represented by ASV1 and ASV2).

## Author Contributions

AG, RQ, and ED conceived and supervised the study. RU and PC carried out the acclimatization, adaptation, and domestication experiments. RU, AM-B, and CR-V carried out the bioinformatic analyses. RU and HN performed the molecular biology experiments. All authors analyzed the data. RQ, RU, and AM-B analyzed and interpreted the data and wrote the paper. All authors read and approved the final manuscript.

## Conflict of Interest Statement

The authors declare that the research was conducted in the absence of any commercial or financial relationships that could be construed as a potential conflict of interest.

## Footnotes

1 www.dsmz.de

2 www.ncbi.nlm.nih.gov

3 https://github.com/widdowquinn/pyani

## References

[B1] Aziz R. K. K. Bartels D. Best A. A. DeJongh M. Disz T. Edwards R. A. (2008). The RAST Server: rapid annotations using subsystems technology. *BMC Genomics* 9:75. 10.1186/1471-2164-9-75 18261238 PMC2265698

[B2] Barrick J. E. Lenski R. E. (2013). Genome dynamics during experimental evolution. *Nat Rev. Genet.* 14 827–839. 10.1038/nrg3564 24166031 PMC4239992

[B3] Bokulich N. A. Kaehler B. D. Rideout J. R. Dillon M. Bolyen E. Knight R. (2018). Optimizing taxonomic classification of marker-gene amplicon sequences with QIIME 2’s q2-feature-classifier plugin. *Microbiome* 6:90. 10.1186/s40168-018-0470-z 29773078 PMC5956843

[B4] Bolger A. M. Lohse M. Usadel B. (2014). Trimmomatic: a flexible trimmer for illumina sequence data. *Bioinformatics* 30 2114–2120. 10.1093/bioinformatics/btu170 24695404 PMC4103590

[B5] Bolyen E. Rideout J. R. Dillon M. R. Bokulich N. A. Abnet C. Al-Ghalith G. A. (2018). QIIME 2: reproducible, interactive, scalable, and extensible microbiome data science. *PeerJ* 6:e27295v1 10.7287/peerj.preprints.27295v1 PMC7015180 31341288

[B6] Brune K. D. Bayer T. S. (2012). Engineering microbial consortia to enhance biomining and bioremediation. *Front. Microbiol.* 3:203. 10.3389/fmicb.2012.00203 22679443 PMC3367458

[B7] Bryan C. G. Joulian C. Spolaore P. Challan-Belval S. El Achbouni H. Morin D. (2009). Adaptation and evolution of microbial consortia in a stirred tank reactor bioleaching system: indigenous population versus a defined consortium. *Adv. Mater. Res.* 71 79–82. 10.4028/www.scientifi.net/AMR.71-73.79

[B8] Bryan C. G. Joulian C. Spolaore P. El Achbouni H. Challan-Belval S. Morin D. (2011). The efficiency of indigenous and designed consortia in bioleaching stirred tank reactors. *Min. Eng.* 24 1149–1156. 10.1016/j.mineng.2011.03.014

[B9] Callahan B. J. McMurdie P. J. Rosen M. J. Han A. W. Johnson A. J. A. Holmes S. P. (2016). DADA2: high-resolution sample inference from Illumina amplicon data. *Nat. Methods* 13 581–583. 10.1038/nmeth.3869 27214047 PMC4927377

[B10] Caporaso J. G. Lauber C. L. Walters W. A. Berg-Lyons D. Lozupone C. A. Turnbaugh P. J. (2011). Global patterns of 16S rRNA diversity at a depth of millions of sequences per sample. *Proc. Natl. Acad. Sci. U.S.A.* 15 4516–4522. 10.1073/pnas.1000080107 20534432 PMC3063599

[B11] Contreras-Moreira B. Vinuesa P. (2013). GET_HOMOLOGUES, a versatile software package for scalable and robust microbial pangenome analysis. *Appl. Environ. Microbiol.* 79 7696–7770. 10.1128/AEM.02411-13 24096415 PMC3837814

[B12] Douglas G. L. Klaenhammer T. R. (2010). Genomic evolution of domesticated microorganisms. *Annu. Rev. Food Sci. Technol.* 1 397–414. 10.1146/annurev.food.102308.124134 22129342

[B13] Edwards K. J. Gihring T. M. Banfield J. F. (1999). Seasonal variations in microbial populations and environmental conditions in an extreme acid mine drainage environment. *Appl. Environ. Microbiol.* 65 3627–3632. 10427059 10.1128/aem.65.8.3627-3632.1999 PMC91544

[B14] Eren A. M. Maignien L. Sul W. J. Murphy L. G. Grim S. L. Morrison H. G. (2013). Oligotyping: differentiating between closely related microbial taxa using 16S rRNA gene data. *Methods Ecol. Evol.* 4 1111–1119. 10.1111/2041-210X.12114 24358444 PMC3864673

[B15] Franzmann P. D. Haddad C. M. Hawkes R. B. Robertson W. J. Plumb J. J. (2005). Effects of temperature on the rates of iron and sulfur oxidation by selected bioleaching Bacteria and Archaea: application of the Ratkowsky equation. *Miner. Eng.* 18 1304–1314. 10.1016/j.mineng.2005.04.006

[B16] Gallone B. Mertens S. Gordon J. L. Maere S. Verstrepen K. J. Steensels J. (2018). Origins, evolution, domestication and diversity of *Saccharomyces* beer yeasts. *Curr. Opin. Biotechnol.* 49 148–155. 10.1016/j.copbio.2017.08.005 28869826

[B17] Giaveno A. Lavalle L. Chiacchiarini P. Donati E. R. (2007). “Airlift Reactors: characterization and applications in Biohydrometallurgy,” in *Microbial Processing of Metal Sulfides*, eds Donati E. R. Sand W. (Berlin: Springer), 169–191. 10.1007/1-4020-5589-7

[B18] Gomez J. M. Caro I. Cantero D. (1996). Kinetic equation for growth of *Thiobacillus ferrooxidans* in submerged culture over aqueous ferrous sulphate solutions. *J. Biotechnol.* 48 147–152. 10.1016/0168-1656(96)01504-0

[B19] Goris J. Konstantinidis K. T. Klappenbach J. A. Coenye T. Vandamme P. Tiedje J. M. (2007). DNA-DNA hybridization values and their relationship to whole-genome sequence similarities. *Int. J. Syst. Evol. Microbiol.* 57 81–91. 10.1099/ijs.0.64483-0 17220447

[B20] Harahuc L. Lizama H. M. Suzuki I. (2000). Selective Inhibition of the oxidation of ferrous iron or sulfur in *Thiobacillus ferrooxidans*. *Appl. Environ. Microbiol.* 66 1031–1037. 10.1128/AEM.66.3.1031-1037.2000 10698768 PMC91939

[B21] Harrison S. T. L. (2016). “Biotechnologies that utilise acidophiles,” in *Acidophiles: Life in Extremely Acidic Environments*, eds Quatrini R. Johnson D. B. (Poole: Caister Academic Press), 265–284. 10.21775/9781910190333.16

[B22] Hedrich S. Gueìzennec A. G. Charron M. Schippers A. Joulian C. (2016). Quantitative monitoring of microbial species during bioleaching of a copper concentrate. *Front. Microbiol.* 7:2044. 10.3389/fmicb.2016.02044 28066365 PMC5167697

[B23] Hedrich S. Schlömann M. Johnson D. B. (2011). The iron-oxidizing proteobacteria. *Microbiology* 157 1551–1564. 10.1099/mic.0.045344-0 21511765

[B24] Hong J. Silva R. A. Park J. Lee E. Park J. Kim H. (2016). Adaptation of a mixed culture of acidophiles for a tank biooxidation of refractory gold concentrates containing a high concentration of arsenic. *J. Biosci. Bioeng.* 121 536–542. 10.1016/j.jbiosc.2015.09.009 26481159

[B25] Johnson D. B. Kanao T. Hedrich S. (2012). Redox transformations of iron at extremely low ph: fundamental and applied aspects. *Front. Microbiol.* 3:96. 10.3389/fmicb.2012.00096 22438853 PMC3305923

[B26] King R. J. (2002). Arsenopyrite. *Geol.Today* 18 72–78. 10.1046/j.1365-2451.2002.t01-1-00006.x

[B27] Kolthoff J. M. Sandell E. B. (1963). *Textbook of Quantitative Inorganic Chemistry.* New York, NY: MacMillan Publishing Co.

[B28] Langmead B. Salzberg S. (2012). Fast gapped-read alignment with Bowtie 2. *Nat. Methods* 9 357–359. 10.1038/nmeth.1923 22388286 PMC3322381

[B29] Liu J. Hua Z. S. Chen L. X. Kuang J. L. Li S. J. Shu W. S. (2014). Correlating microbial diversity patterns with geochemistry in an extreme and heterogeneous environment of mine tailings. *Appl. Environ. Microbiol.* 80 3677–3686. 10.1128/AEM.00294-14 24727268 PMC4054149

[B30] Makarova K. Slesarev A. Wolf Y. Sorokin A. Mirkin B. Koonin E. (2006). Comparative genomics of the lactic acid bacteria. *Proc. Natl. Acad. Sci. U.S.A.* 103 15611–15616. 10.1073/pnas.0607117103 17030793 PMC1622870

[B31] McMurdie P. J. Holmes S. (2013). Phyloseq: an r package for reproducible interactive analysis and graphics of microbiome census data. *PLoS One* 8:e61217. 10.1371/journal.pone.0061217 23630581 PMC3632530

[B32] Meier-Kolthoff J. P. Auch A. F. Klenk H. P. Göker M. (2013). Genome sequence-based species delimitation with confidence intervals and improved distance functions. *BMC Bioinformatics* 14:60. 10.1186/1471-2105-14-60 23432962 PMC3665452

[B33] Meier-Kolthoff J. P. Hahnke R. L. Petersen J. Scheuner C. Michael V. Fiebig A. (2014). Complete genome sequence of DSM 30083T, the type strain (U5/41T) of *Escherichia coli*, and a proposal for delineating subspecies in microbial taxonomy. *Stand. Genomic Sci.* 9:2. 10.1186/1944-3277-9-2 25780495 PMC4334874

[B34] Méndez-García C. Mesa V. Sprenger R. R. Richter M. Diez M. S. Solano J. (2014). Microbial stratification in low pH oxic and suboxic macroscopic growths along an acid mine drainage. *ISME J.* 8 1259–1274. 10.1038/ismej.2013.242 24430486 PMC4030236

[B35] Merodio J. C. Martínez J. M. (1985). Análisis químico de componentes mayoritarios en rocas silicatadas. *Rev. Asoc. Arg. Min. Petrol.* 16 7–16.

[B36] Mutch L. A. Watling H. R. Watkin E. L. J. (2010). Microbial population dynamics of inoculated low-grade chalcopyrite bioleaching columns. *Hydrometallurgy* 104 391–339. 10.1016/j.hydromet.2010.02.022

[B37] Namiki T. Hachiya T. Tanaka H. Sakakibara Y. (2012). MetaVelvet: an extension of Velvet assembler to *de novo* metagenome assembly from short sequence reads. *Nucleic Acids Res.* 40:e155. 10.1093/nar/gks678 22821567 PMC3488206

[B38] Nieto P. A. Covarrubias P. C. Jedlicki E. Holmes D. S. Quatrini R. (2009). Selection and evaluation of reference genes for improved interrogation of microbial transcriptomes: case study with the extremophile *Acidithiobacillus ferrooxidans*. *BMC Mol. Biol.* 10:63. 10.1186/1471-2199-10-63 19555508 PMC2713239

[B39] Norris P. (2007). “Acidophile diversity in mineral sulfide oxidation,” in *Biomining*, eds Rawlings D. E. Johnson D. B. (Berlin: Springer-Verlag), 199–216. 10.1007/978-3-540-34911-2_10

[B40] Nuñez H. Moya-Beltrán A. Covarrubias P. C. Issotta F. Cárdenas J. P. González M. (2017). Molecular systematics of the genus *Acidithiobacillus*: insights into the phylogenetic structure and diversification of the taxon. *Front. Microbiol.* 8:30 10.3389/fmicb.2017.00030 PMC5243848 28154559

[B41] Okibe N. Gericke M. Hallberg K. B. Johnson D. B. (2003). Enumeration and characterization of acidophilic microorganisms isolated from a pilot plant stirred tank bioleaching operation. *Appl. Environ. Microbiol.* 69 1936–1943. 10.1128/AEM.69.4.1936-1943.2003 12676667 PMC154788

[B42] Okibe N. Johnson D. B. (2004). Biooxidation of pyrite by defined mixed cultures of moderately thermophilic acidophiles in pH-controlled bioreactors: significance of microbial interactions. *Biotechnol. Bioeng.* 87 574–583. 10.1002/bit.20138 15352055

[B43] Quatrini R. Appia-Ayme C. Denis Y. Jedlicki E. Holmes D. S. Bonnefoy V. (2009). Extending the models for iron and sulfur oxidation in the extreme acidophile *Acidithiobacillus ferrooxidans*. *BMC Genomics* 10:394. 10.1186/1471-2164-10-394 19703284 PMC2754497

[B44] Quatrini R. Johnson D. B. (2018). Microbiomes in extremely acidic environments: functionalities and interactions that allow survival and growth of prokaryotes at low pH. *Curr. Opin. Microbiol.* 43 139–147. 10.1016/j.mib.2018.01.011 29414445

[B45] Quatrini R. Ossandon F. J. Rawlings D. E. (2016). “The flexible genome of acidophilic prokaryotes,” in *Acidophiles: Life in Extremely Acidic Environments*, eds Quatrini R. Johnson D. B. (Poole: Caister Academic Press), 199–220. 10.21775/9781910190333.12

[B46] Raes J. Korbel J. O. Lercher M. J. von Mering C. Bork P. (2007). Prediction of effective genome size in metagenomic samples. *Genome Biol.* 8:R10. 10.1186/gb-2007-8-1-r10 17224063 PMC1839125

[B47] Rawlings D. E. (2005). Characteristics and adaptability of iron- and sulfur-oxidizing microorganisms used for the recovery of metals from minerals and their concentrates. *Microb. Cell Fact.* 4:13. 10.1186/1475-2859-4-13 15877814 PMC1142338

[B48] Rawlings D. E. (2007). “Relevance of cell physiology and genetic adaptability of biomining microorganisms to industrial processes,” in *Biomining*, eds Rawlings D. E. Johnson D. B. (Berlin: Springer-Verlag), 77–198. 10.1007/978-3-540-34911-2_9

[B49] Rawlings D. E. Dew D. du Plessis C. (2003). Biomineralization of metal containing ores and concentrates. *Trend Biotechnol.* 21 38–44. 10.1016/S0167-7799(02)00004-5 12480349

[B50] Rawlings D. E. Johnson D. B. (2007). The microbiology of biomining: development and optimization of mineral-oxidizing microbial consortia. *Microbiology* 153 315–324. 10.1099/mic.0.2006/001206-0 17259603

[B51] Rawlings D. E. Silver S. (1995). Mining with microbes. *Nat. Biotech.* 13 773–778. 10.1038/nbt0895-773

[B52] Rawlings D. E. Tributsch H. Hansford G. S. (1999). Reasons why ’*Leptospirillum*’-like species rather than *Thiobacillus ferrooxidans* are the dominant iron-oxidizing bacteria in many commercial processes for the biooxidation of pyrite and related ores. *Microbiology* 145 5–13. 10.1099/13500872-145-1-5 10206710

[B53] Reith F. Lengke M. F. Falconer D. Craw D. Southam G. (2007). The geomicrobiology of gold. *ISME J.* 1 567–584. 10.1038/ismej.2007.75 18043665

[B54] Rognes T. Flouri T. Nichols B. Quince C. Mahé F. (2016). VSEARCH: a versatile open source tool for metagenomics. *Peer J.* 4:e2584. 10.7717/peerj.2584 27781170 PMC5075697

[B55] Silverman M. P. Lundgren D. G. (1959). Studies on the chemoautotrophic iron bacterium *Ferrobacillus*. *J. Bacteriol.* 78 326–331. 14446765 10.1128/jb.78.3.326-331.1959 PMC290544

[B56] Spolaore P. Joulian C. Gouin J. Morin D. d’ Hugues P. (2010). Relationship between bioleaching performance, bacterial community structure and mineralogy in the bioleaching of a copper concentrate in stirred-tank reactors. *Appl. Microbiol. Biotechnol.* 89 441–448. 10.1007/s00253-010-2888-5 20890755

[B57] Suzuki I. Lee D. Mackay B. Harahuc L. Oh J. K. (1999). Effect of various ions, pH, and osmotic pressure on oxidation of elemental sulfur by *Thiobacillus thiooxidans*. *Appl. Environ. Microbiol.* 65 5163–5168. 10543839 10.1128/aem.65.11.5163-5168.1999 PMC91697

[B58] Tischler J. S. Jwair R. J. Gelhaar N. Drechsel A. Skirl A.-M. Wiacek C. (2013). New cultivation medium for “*Ferrovum*” and *Gallionella*-related strains. *J. Microbiol. Methods* 95 138–144. 10.1016/j.mimet.2013.07.027 23954479

[B59] Vogel A. I. (1989). *Vogel’s Textbook of Quantitative Chemical Analysis*, 5th Edn London: Longman Group Ltd.

[B60] Wakeman K. Auvinen H. Johnson D. B. (2008). Microbiological and geochemical dynamics in simulated heap leaching of a polymetallic sulfide ore. *Biotechnol. Bioeng.* 101 739–750. 10.1002/bit.21951 18496880

[B61] Wang Y. Zeng W. Qiu G. Chen X. Zhou H. (2014). A moderately thermophilic mixed microbial culture for bioleaching of chalcopyrite concentrate at high pulp density. *Appl. Environ. Microbiol.* 80 741–750. 10.1128/AEM.02907-13 24242252 PMC3911102

