# Supplementary figures and images for: Domestication of Local Microbial Consortia for Efficient Recovery of Gold Through Top-Down Selection in Airlift Bioreactors
Source: Front Microbiol. 2019 Jan 30;10:60. doi: 10.3389/fmicb.2019.00060 (PMC6363673; doi:10.3389/fmicb.2019.00060)

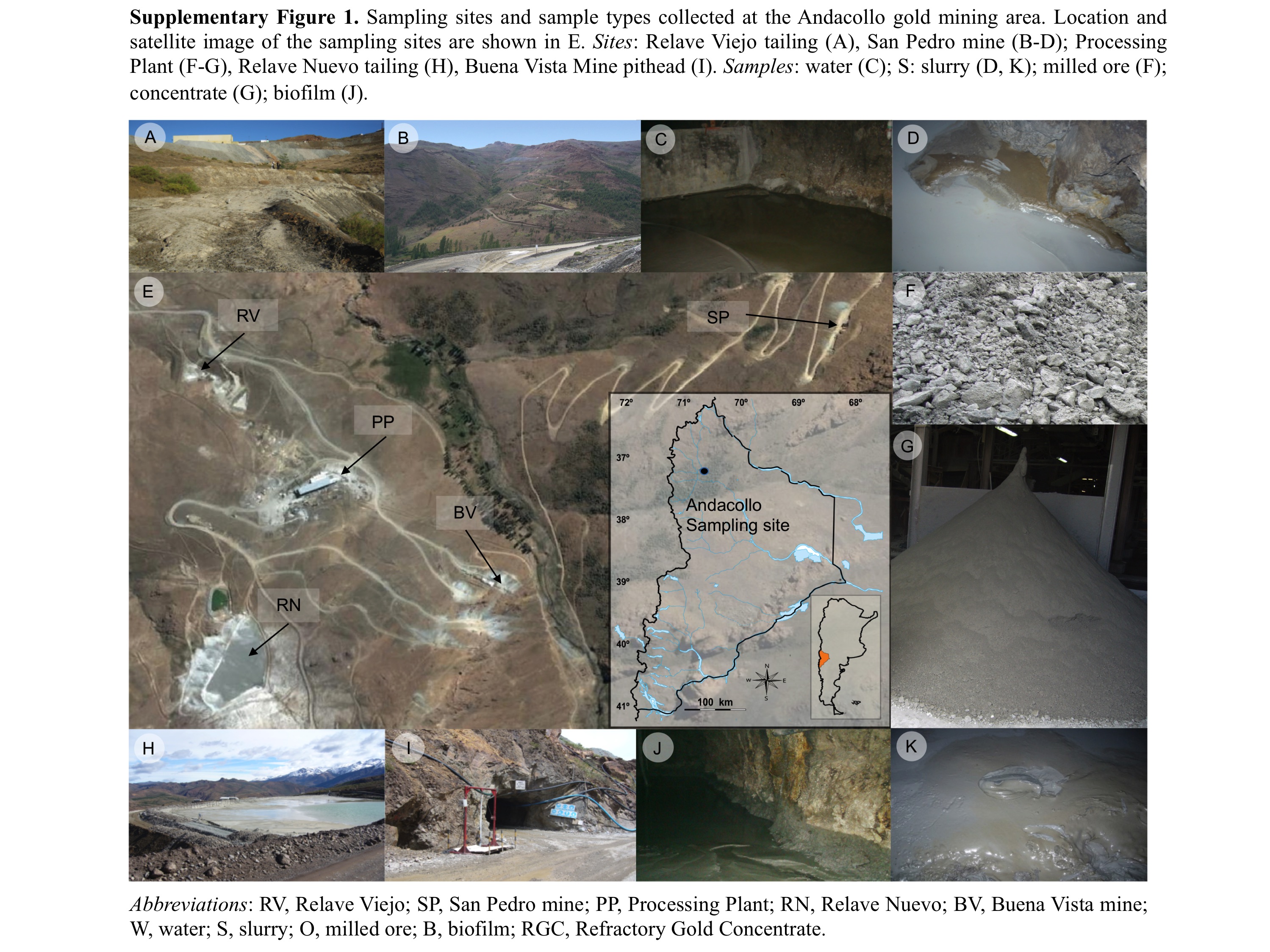

Supplement: Supplementary file 6 [file Image_1.jpg]

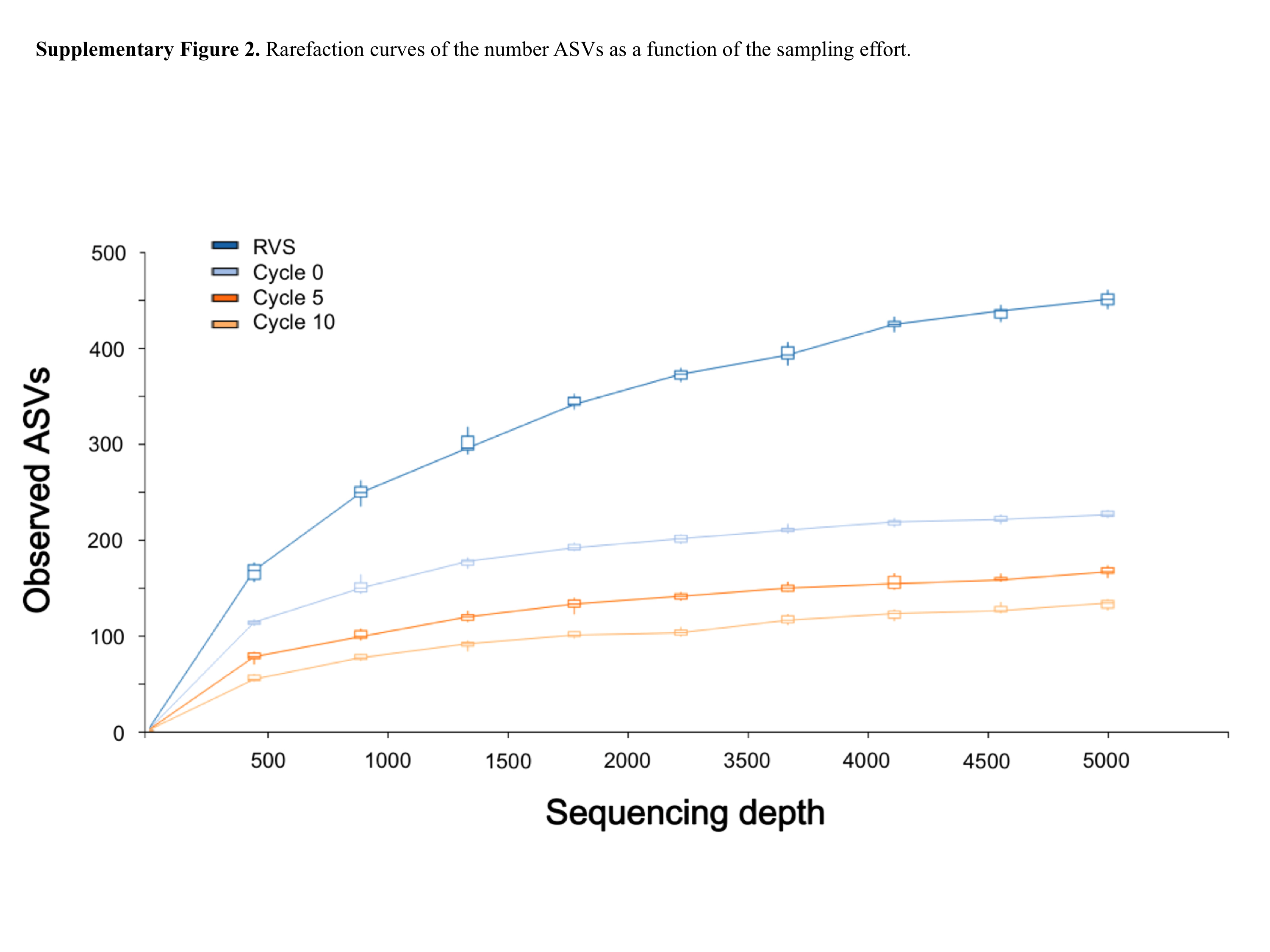

Supplement: Supplementary file 7 [file Image_2.jpg]

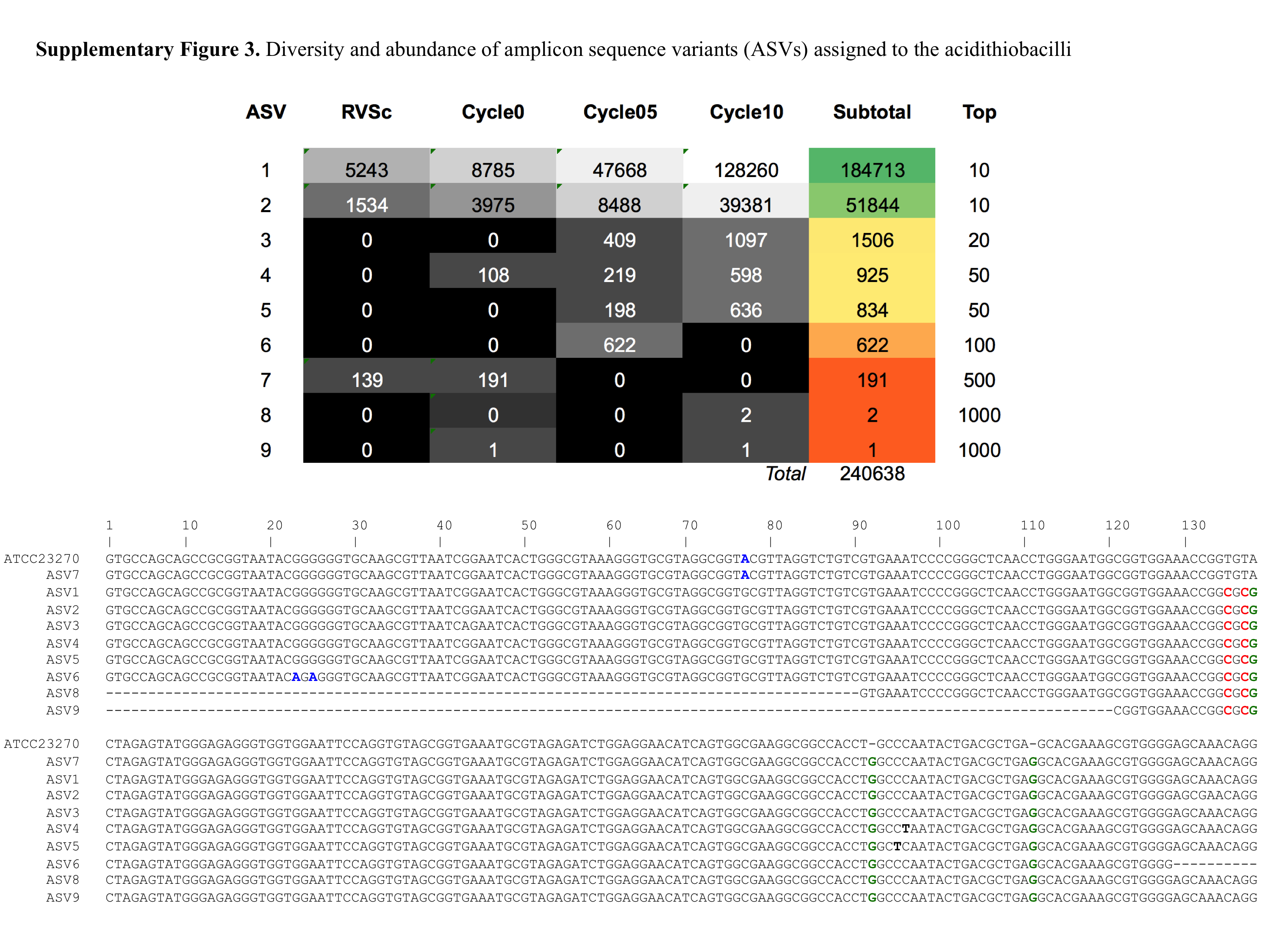

Supplement: Supplementary file 8 [file Image_3.jpg]

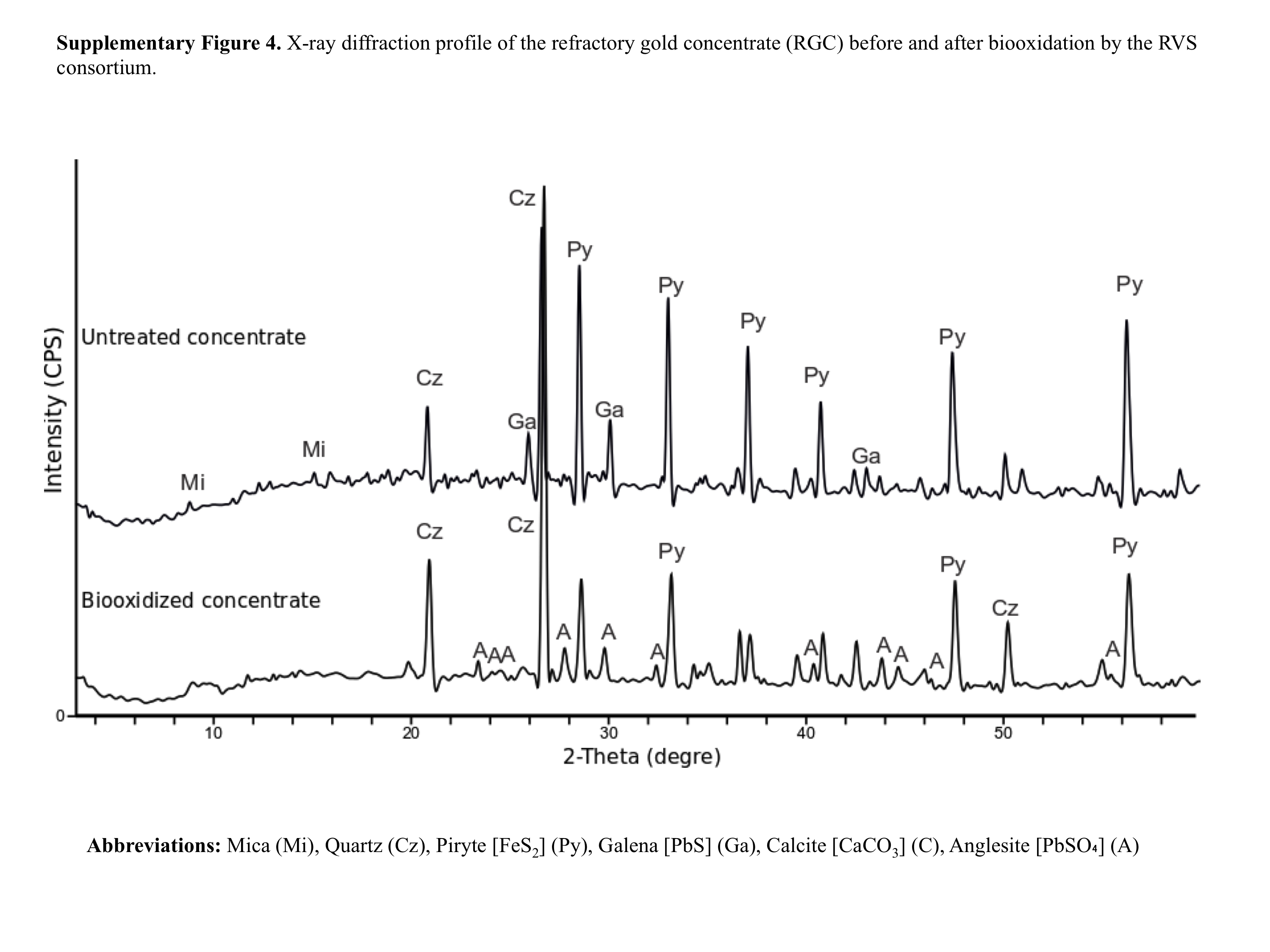

Supplement: Supplementary file 9 [file Image_4.jpg]
